# BioMEMS –Advancing the Frontiers of Medicine

**DOI:** 10.3390/s8096077

**Published:** 2008-09-26

**Authors:** Teena James, Manu Sebastian Mannoor, Dentcho V. Ivanov

**Affiliations:** 1 Microelectronics Research Center and New Jersey Institute of Technology, Newark, NJ, U.S.A.; E-mail: msm28@njit.edu (M. S. M.);; 2 Dept of Biomedical Engineering, New Jersey Institute of Technology, Newark, NJ, U.S.A.; E-mail: msm28@njit.edu (M. S. M.);

**Keywords:** BioMEMS, Microtechnology, Medical applications

## Abstract

Biological and medical application of micro-electro-mechanical-systems (MEMS) is currently seen as an area of high potential impact. Integration of biology and microtechnology has resulted in the development of a number of platforms for improving biomedical and pharmaceutical technologies. This review provides a general overview of the applications and the opportunities presented by MEMS in medicine by classifying these platforms according to their applications in the medical field.

## 1. Introduction

Micro-electro-mechanical-systems (MEMS) were introduced in the late 80s as an extension of the traditional semiconductor very-large-scale-integration (VLSI) technologies. MEMS technology adapted for biological and medical applications emerged into a new field of research – BioMEMS, or Bio Micro Electro Mechanical Systems. Over the last decades, this technology has led to significant advances in different fields of medicine and biology through the development of a variety of micro engineered device architectures. This acceleration is primarily due to the fact that microfabrication technology provides device miniaturization, as well as better performance, lower cost and higher reliability.

Although an exhaustive review of all the biomedical applications of MEMS technology cannot be done due to the large number of rapid advances in this field, in this review, we try to identify and discuss some of the major areas in medical industry that have profited from the development of MEMS.

## 2. MEMS Technology

In order to understand how the MEMS technique is utilized for the development of devices with potential biomedical applications, a primary understanding of the standard micro-fabrication processes is necessary. MEMS technology can basically be described as the development of device structures in the micro- or even nano-dimensions using “micromachining process” on silicon and other substrates. By utilizing the peculiar characteristics of the material silicon, complex micro 3D structures such as channels, pyramids or V-grooves can be formed. The processes used for carrying out such “micro scale” fabrication can mainly be classified into patterning process, material removal processes and material deposition processes.

### 2.1. Patterning Technique

**Figure 1:**
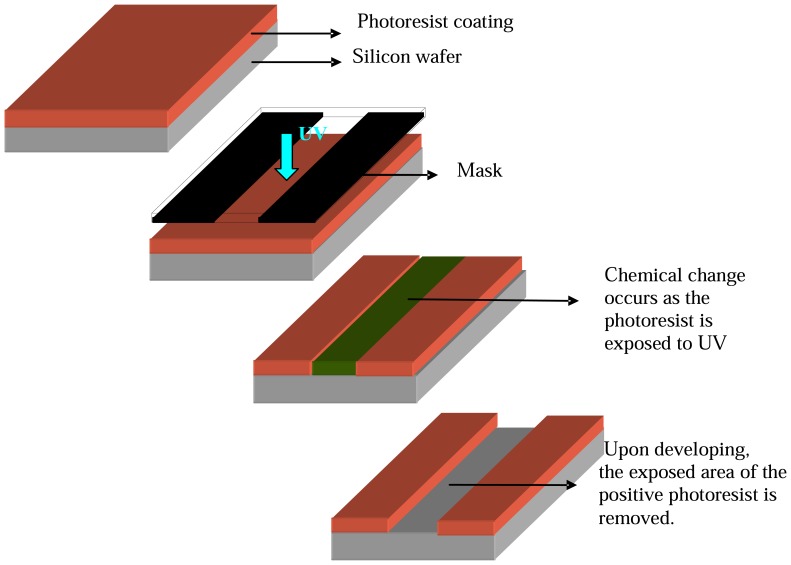
Illustration of MEMS photolithographic process.

This is the process of transferring the desired patterns onto a wafer surface. In MEMS lithography processes, the wafer is first coated with a photosensitive material known as photoresist and then exposed to radiation source such as UV through a mask which contains the pattern that has to be transferred on to the wafer surface. There are two types of photoresist, positive and negative. According to the type of photoresist used, the exposed area is either retained or removed after development (chemical treatment using ‘developer’). This process thus opens up ‘windows’ into the underlying material and serves as a mask for further processing. Once this mask has served its purpose the photoresist is stripped off using chemical treatment. The MEMS lithographic procedure is illustrated below (Figure 1).

### 2.2. Material Removal Processes

**Figure 2:**
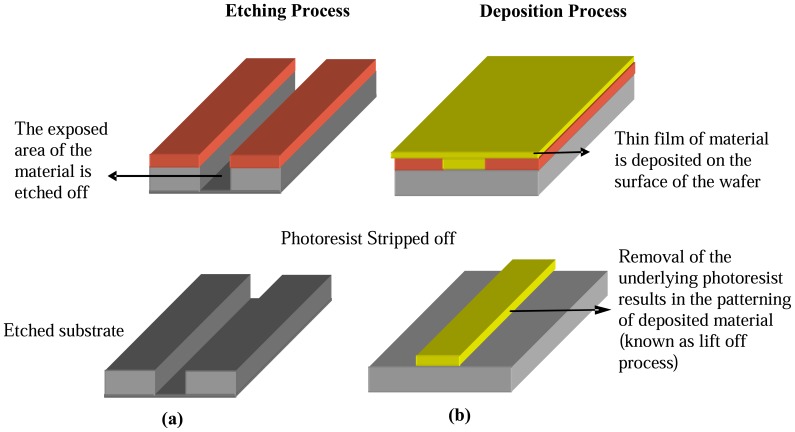
Illustration of Etching and Deposition process in MEMS technology

Selective removal of substrate or materials is carried out by the process known as etching. The dissolving power of a chemical solution is utilized to remove the material in wet etching. Selective etching is done by covering the desired portions of the material with a mask that resist the dissolution power of the solution as shown in Figure 2a. In dry etching, the material is removed by bombardment with high energy ions. The most commonly used methods in dry etching are Reactive ion etching (RIE) and sputter etching. The material is removed as a result of chemical reaction with the ions in RIE whereas in sputter etching there is no chemical reaction involved and the material atoms are knocked off due to the high energy ions. For the purpose of etching deeper, Deep Reactive Ion Etching (DRIE) is used which is an extension of Reactive Ion Etching.

### 2.3. Material Deposition Processes

In this process a thin layer of material is deposited on the surface of the wafer as shown in Fig 2b. According the various physical and chemical properties of the material being deposited, different processes exists for deposition. They can be classified mainly as chemical deposition; wherein chemical reactions are required for the formation of new molecules and physical deposition where no new molecules are made but are taken from a source and deposited on the surface of the substrate. In Low pressure Chemical Vapor Deposition Processes (LPCVD) process, materials are deposited at low pressure. Although this process produces a more uniform layer of material, high temperature is required and thus this process may prove unsuitable in certain situations. Plasma Enhanced Chemical Vapor Deposition(PECVD) allows reactions to happen at lower temperature as the gases are ionized by plasma with the drawback that the thin films produced by this processes is of poorer quality. Molecular Beam Epitaxial growth is a relatively new technique where films of molecular thickness are deposited.

Having discussed briefly about the MEMS techniques, we shall now look into how devices fabricated using the above mentioned processes have revolutionized the world of Medicine. The application of MEMS technology is very broad, and it is almost impossible to discuss all the various researches done in this direction. However, we have tried to classify most of the advances under four broad titles- Diagnostics, Therapeutics, Prosthesis, and Surgery.

## 3. Diagnostics

Diagnostics have always been an indispensible component of healthcare. As science and technology progressed over the years, diagnostics have also been subjected to continuous improvement. Currently much research and development are put into the development of personalized diagnostic tools that are highly sensitive and capable of early detection of diseases. Consumer friendly, at home pregnancy tests and hand held glucose monitoring systems are some of the substantial diagnostic advancement that has occurred during the last few years. MEMS capabilities are incorporated for developing such hand held diagnostic equipments where laboratory analysis has to be carried out in miniaturized scale.

Diagnostics can be broadly classified into five major segments as shown and MEMS technology has profited all these different areas by providing sensing platforms capable of detecting these analytes. A summary of these are given in the table.

### 3.1. Point of care diagnostics

The idea of downsizing diagnostic tools was made a reality by the engineering advances in surface and material science. In order to meet the need for more integrated and immediate clinical results, Micro-electromechanical systems (MEMS) use techniques akin to those used in the microelectronic industry to build these miniaturized devices that are capable of purifying, isolating and characterizing samples, thus putting the entire assaying operation on a single chip. These systems are also known as μTAS (micro Total Analysis Systems) or Lab-on-a-Chip devices. Such a platform integrates sample treatment modules together with separation and detection modules all brought together using the functionality of MEMS techniques. The potential for such devices are many including detection of biological weapons through protein and DNA analysis, blood analysis and drug screening systems.

For the determination of a specific type of disease, multiple multiplexing of tests might be required. Each test assay might have different stages and each stage might use a different MEMS component. We review the various MEMS-based components that could be applied to the different stages of the assaying operation such as cell separation, cell lysis, analyte molecule purification and sensing. For detection of various types of diseases, different biomedical specimen has to be used. Using well designed microfluidic processors together with other MEMS components, it is possible to isolate and analyze a particular type of biomolecule from the specimen. This requires a number of stages such as sample preparation stages and measurement stages.

#### 3.1.1. Cell Sorting

Using MEMS technology it is possible to isolate cells based on their physical and chemical properties from a large population of heterogeneous cells [43]. MEMS structures based on phenomena such as hydrodynamics, optodynamics and electro kinetics have been used for this purpose. Structures using hydrodynamics take advantage of the laminar hydrodynamic focusing used in flow cytometry for the sorting of cells. Although this provides good sorting results, external activation devices such as pumps are required for high pressure fluid manipulation. In dielectrophorosis, the cells exhibit dielectrophoretic activity in the presence of an external electric field thus making it possible for them to be manipulated and separated. Using this method, MDA231 cancer cells have been separated from dilute blood by selective capture onto microelectrodes [44]. Single cell capture and manipulation has also been shown to be possible using this technique. In another technique using electric field – electrophoresis, individual cells are moved due to their intrinsic charges. Studies have shown that application of an electric field across a thin micro porous membrane, results in the trapping of cells on the membrane [45] Other techniques using ligand proteins immobilized on surface of channels and wells can also be used to trap cells in microdevices [46, 47]. Optical tweezers have also been applied for cell sorting by transferring the momentum from a focused laser beam on to cells [48-50]. Using this technique, differentiation of cancer cells from normal cells has been demonstrated [51]. Yasuda and colleagues used this method together with an on-chip microculture chip, for comparing genetically identical cells [52].

#### 3.1.2. Cell Lysis

Analysis of intracellular materials for studying physiologic and pathologic condition typically requires cell lysis. Devices that perform cell lysis requires speed (to prevent further biochemical changes), selectivity (breaking down cell membranes while protecting organelle membranes), and integration with other microfluidic devices [53]. Thermal lysing method, probably due to its compatibility with PCR (polymerase chain reaction) is one of the most commonly used lysing mechanism [54]. In this method, the cell membrane breaks down as a result of high temperature provided by the micro heaters. Although this technique is well suited for DNA analysis and detection, the denaturization of proteins at elevated temperatures is a major concern. For overcoming this limitation, Marentis et al [55] used energy of sound wave from a piezoelectric minisonicator to lyse leukemia HL-60 cells and Bacillus subtilis bacterial spores in microfluidic environment. Marmottant et al introduced the novel idea of using oscillating micro bubbles to rupture cell membranes[56]. Other microscale mechanisms have also been developed such as nanoscale barbs [57] through which cells are forced causing them to rupture, and electrical lysing [58, 53, 59] wherein cells are subjected to high electric fields causing the cell membrane to destabilize and thus rupture.

#### 3.1.3. Purification

Once the cell has been disrupted, enrichment of analyte molecules (proteins and nucleic acids) might be required before analysis. MEMS based electrophoresis and isoelectric focusing [60, 61] have been used for this purpose. The intrinsic charges of the biomolecules are utilized in these methods. Specially treated bead filled columns for nucleic acid or protein purification can also be incorporated into the chip. Another alternative include polyimide membrane coated microfluidic channel capable of absorbing the specific analytes.

Amplification of nucleic acids can be done using polymerase chain reaction (PCR). The first microPCR was reported by Northrup *et al.* [62] in 1995. This device basically consisted of polysilicon miniature heaters. Since then, much effort had been made to improve this device and currently there are many commercial mini PCR systems. A number of literatures are available on various MEMS based PCR devices [63-67]. An excellent review of the technology for MEMS PCR is given by Zhang et al [68]. Nucleic acids and proteins after purification can be fractioned using nano-sieves[69].

#### 3.1.4. Molecular Biosensors

Today, many molecular based diagnostics are emerging that enable identification of susceptibility to diseases long before the actual symptoms are manifested. Protein expression and genetic makeup of cells can reveal a great amount of information which can be useful in innumerable ways for medical purposes. Currently, much research has been done in this area for developing sensors capable of identifying specific protein molecules and nucleotide sequences.

Several techniques exist for the detection and quantification of proteins and nucleic acids. These detection schemes can be classified into two categories -*labeled* and *label free* methods. Although labeled methodology offers more sensitivity, the labeling procedure is both time consuming and expensive. Therefore, studies have focused more on the development of label-free detection techniques.

The following section briefly describes the basic sensing principles used in the various MEMS. based biosensing structures. Most of the biosensors are *affinity*-*based*, which uses a biorecognition layer (probe molecules) immobilized on a transducer surface to bind to the analyte molecules selectively. Microfabricated biosensors utilizing electrical, mechanical, piezoelectric and acoustic signal transduction mechanisms have been developed over the years. Along with this, biosensors based on nanotubes and nanowires have also been developed recently due to the advent of nanotechnology.

The most common electrical device architectures used for biosensing include micro fabricated capacitive electrodes and field effect transitive structures. Mechanical sensing structures such as micro-cantilevers [70-75] and diaphragms have also been shown to be extremely sensitive to biomolecular interactions and has currently been shown to weigh a single molecule [76]. The variations in mass as a result of target-probe binding are detected by these structures. Acoustic transducing mechanism is another major method that has been used for the detection of biomolecules. Changes in wave properties caused by biomolecular interactions at the surface of the sensor are detected by these sensors. Various surface acoustic waves can propagate on the surface a piezoelectric substrate, and according to them, various sensor configurations exist of biomolecular detection.

### 3.2. Other MEMS based diagnostics

Apart from the above mentioned devices, other remarkable advances of BioMEMS include development of devices for measuring physiological parameters such as temperature, pressure, pulse rate etc. Intra-ocular [77-80], intra-cranial [81-84] and cardio-vascular pressure sensors [85-91] have been developed using MEMS techniques. Other MEMS applications include sensors embedded into smart textiles or wearable cardiovascular monitoring systems, such as wearable ECG foils [92].

## 4. Therapeutic Applications

Under this heading, we will review the MEMS contribution in small molecule therapeutics i. e. drug discovery, drug screening, and drug delivery. To start with, a small introduction about the conventional drug screening procedure seems necessary.

### 4.1. Drug Discovery

Drug discovery process is organized into different phases starting with the identification of drug targets, a process known as target identification, wherein the biomolecules that play significant role in diseases are identified. Next, from a library of innumerable number of chemical compounds, the ones that have the potential to treat the disease by interacting with the drug targets in a desirable way are identified. This process is known as lead identification. These compounds undergo optimization to become a possible clinical candidate followed by a testing phase to ensure that it is safe to be administered to the patients. The advances in drug discoveries are highly dependent on the technological advancement made in these stages. MEMS technology has penetrated into many of these stages to make it easier to find refine and test possible drug candidates.

#### 4.1.1. Target Identification

For disease target identification, the etiology (causative factor) of disease has to be known and the molecular machinery behind the abnormal/malfunctioning biological processes has to be determined. The collective contribution of studies on genetic interaction (genomics), protein expressions and interactions (proteomics) and their relation to diseases allows for the rapid and precise discovery of these drug targets. Genomics has taken a huge leap by the advent of high throughput technologies like microarrays. Microarray generally applies to spots with diameters of 200 microns or less attached to a solid surface such as a silicon chip thus allowing larger-scale experiments using very small volumes of sample and reagents.

In order to understand the application of MEMS in this area, a basic discussion about the target identification process is necessary. For studying the gene expression of a cell using microarray, the RNA of the cell is extracted and its labeled DNA copies are made. These tagged DNA copies are washed over a microarray containing single stranded DNA with known sequence (probe DNA). Upon finding a complementary probe sequence on the microarray, the tagged single stranded DNA hybridizes with it. Scanning the microarray with a laser source causes the tagged bound DNA to florescence. Since the location and sequence of the probe DNAs are known ahead of time, a comparison of the spots on the microarray reveals the gene expression of the cell.

In microarrays, the number of features or samples on a single slide or array can exceed tens or even hundreds of thousands. Microarrays, created using photolithographic method have extended the capability of the bioassays by reducing its development time and cost thus resulting in enhanced throughput. The high density Gene Chip probe arrays of Affymetrix, developed by Steven Foder and colleagues, contains thousands of oligonucleotides in a very small area of 2cm2 developed by light directed oligonucleotide synthesis (in situ synthesis) [93]. Microelectronic Array devices developed by M.J. Heller of Nanogen is yet another example of the improvement brought about by MEMS techniques. These active electronic microarrays provide electronic addressing of probes and increased DNA hybridization rate through the application of appropriate electric field [94]. Several protein chip formats have also been developed due to the advancement of micro fabrication technologies [95-101].

#### 4.1.2. Lead Identification & optimization

MEMS technology has begun to provide unique tools that can enable earlier determination of lead compounds than what was traditionally possible. Microchip patch clamp is one of such unique tools that have revolutionized the lead identification process [102-105]. High throughput ion channel drug screening is possible using this technique. Patch clamp technique utilizes MEMS functionality to fabricate planar electrodes which are micron sized holes in an insulating layer wherein the cells are trapped and ionic current variations measured.

#### 4.1.3. Preclinical testing

In order to obtain a better idea of drug behavior cellular response studies are very important. Cell based sensors can thus provide functional information about the effects of drugs on its signaling pathways. It is often quite difficult to simulate the actual condition of the environment within a living organism since cells respond to spatially and temporally organized signals in their microenvironment. With MEMS technology it is possible to create microfluidic structures mimicking the actual ‘*in-vivo*’ environment [106-108]. Such micro structures can help in the preclinical testing stage of the drugs as these can create cheap ‘*in-vivo*’ environments.

### 4.2. Drug Delivery

The predominant methods for drug administration such as oral delivery and injection often results in immediate or rapid drug release wherein no control over the rate of drug delivery or the target area of the drug is exercised. A variety of devices and components have been designed and fabricated using MEMS techniques which are able to release drugs of different dosages in different delivery patterns. These include transdermal patches, implants, microparticles and microencapsulation.

#### 4.2.1. Microneedles

Microneedles were first proposed as a method for percutaneous drug delivery in the 1970s [114]. Since then, the microneedle design has been refined to provide better control over drug delivery [115]. Although transdermal drug delivery is one of the most effective modes of administration, the poor permeability of the skin had remained a major limitation for macromolecular drug delivery. In vitro experiments have shown that inserting microneedles into skin can increase permeability by orders of magnitude, thus facilitating transport of therapeutic macromolecules [116]. Needles of micron dimensions can pierce into the skin surface to create holes large enough for molecules to enter, but small enough to avoid pain or significant damage. MEMS technology has made it possible to create such micron sized needles with design considerations dependant on its strength, robustness, minimal insertion pain, and tissue damage in patients.

Various types of microneedles have been fabricated and tested for drug delivery. They exist in two basic designs, in-plane and out-of-plane. In-plane microneedles have openings at the shaft of the needle whereas out-of-plane microneedles have openings at the tip of the needle. In order to avoid the breakage of the top part of the needles inside the skin, microneedle array with biodegradable tips have been developed [117] Encapsulation of molecules within microneedles that dissolve within the skin for bolus or sustained delivery has also been studied [118]. Hollow microneedles have also been fabricated and used to flow drug solutions into the skin [119].

#### 4.2.2. Microreservoirs

For every drug delivery system, a drug depot or supply is required. In the case of in vivo drug delivery system, this drug depot has to protect the drug from the body until needed and when needed has to allow delivery in a controlled manner. Ingestion is widely accepted as the ideal form of drug delivery, but it presents difficulties for a number of newly developed drugs such as proteins and peptides as they are unable to survive the stomach's acidic environment and have reduced bioavailability. In order to overcome this limitation, microparticles and nanoparticles capable releasing drugs at specific targeted areas have been developed [121-125]. T. A Desai and team developed such reservoir-containing silicon micro particles capable of delivering therapeutics to the targeted sites in the gastrointestinal system [124]. The surfaces of these devices were designed to adhere onto specific cells in the digestive tract to deliver drugs by functionalizing them with avidin linked to biotinylated lectins. Various implantable delivery devices have also been developed for targeted and controlled drug delivery. Such a structure was designed and developed by Santini and colleagues, containing an array of individually addressable microreservoirs containing gold membrane, each of which contained a dosage of drug that could be released separately [126, 127]. The implantable drug delivery device developed by MicroCHIPS Inc. is shown below. Other non-traditional MEMS fabrication techniques have also been explored to form reservoirs with greater biocompatibility [128]. Although research on microfabricated drug delivery devices has rapidly expanded in recent years, in order to achieve improved patient compliance, much research still has to be done to optimize the size, shape, number, volume, and surface characteristics of the drug delivery systems.

## 5. Surgical Applications

### 5.1. Minimally Invasive Surgery

The trends in modern medicine are to use less invasive methods that significantly reduces body trauma by performing surgery through smaller incisions using specialized tools. The main advantages of which includes lesser trauma, reduced post-operative pain, and quicker recovery time. MEMS technology has played an important role in the evolution of these minimally invasive procedures. These techniques allow the surgical tools to reach down to the size scale of individual cells and provide access for manipulation in previously inaccessible areas of the body. The development and integration of sensors, actuators and associated electronics required for augmenting the surgeon's dexterity and perception at micron level is possible due to this technology.

#### 5.1.1. Microtools and tactile sensing

Conventional surgical tools have limited capability when it comes to manipulation of small structures such as nerves and vessels of small diameters. In order to achieve higher spatial resolution, researchers have developed tools such as micro-grippers, micro-tweezers, micro-forceps and micro-scissors with the aid of microfabrication techniques [130,131]. Recently, thermally actuated grippers capable of grasping micron sized objects have been developed for use in ophthalmic surgery wherein they could be used within the small volume of the eye [132]. Other development in this area include biocompatible polymer-metal bimorph microforceps made from a sandwich of gold film and SU-8 capable of grasping micron size object such as a single cell [130].

In MIS procedures, a major challenge faced by surgeons is the lack of sense of touch. In order to overcome this limitation microfabricated devices capable of restoring and enhancing tactile sensation is being looked into [133, 134]. MEMS based tactile sensors equipped with the ability to continuously monitor the type of tissue being handled and the force exerted on the tissue has been developed to enhance the surgeon's haptic perception. A miniature tactile sensor array consisting of an array of capacitive sensor cells mounted at the tip of their laparoscopic tool was developed by Gray and Fearing [135]. Deformation of this system by the application of pressure causes a detectable change in the capacitance of the affected cells. In the design for a micro-endoscopic tactile sensor, Rao *et al.* [136] describes a tactile sensor developed using PVDF films for better flexibility and sensitivity. Force sensors typically consisting of piezoelectric or piezoresistive elements embedded at critical locations along the structure of a mechanical device have been developed to provide three-dimensional mapping of the mechanical deformations in the device. In addition to these sensing mechanisms, various other methods have also been investigated. MEMS sensors for monitoring mechanical properties of tissues such as palpitation have also been developed [137]. One of the main research issues to be resolved for the tactile sensor design is the packaging to protect tissue and the sensor and cabling to bring signals out of the body without interfering with its range of motion [138].

#### 5.1.2. MEMS Cutting tools

**Figure 6:**
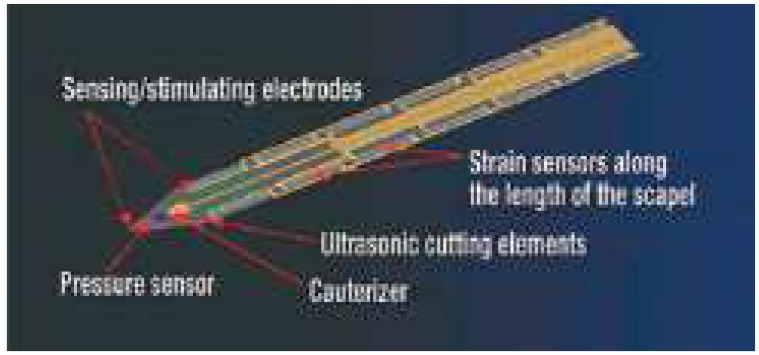
Surgical knife developed by Verimetra Inc.

The principle of miniaturization brings forth ultra small cutting tools which makes smaller incision that causes lesser bleeding. Research has been looking into the development of such nano-knives which are made sharper by etching silicon precisely along its crystal planes [132]. Utilization of vibratory mechanism for cutting tissues have also been demonstrated [139]. A surgical device called data knife developed by Verimetra. Inc (Pittsburg, PA, U.S.A.) is shown in Figure 6. It consists of a scalpel outfitted with different strain sensors along the edges of the blade to sense the amount of force being applied. Vibratory mechanism by piezoelectric actuation is used in this device for cutting through tissues. Other sensing mechanisms included in this device are pressure sensors, temperature sensors as well as impedance sensors for measuring tissue impedance [140].

#### 5.1.3. Endoscopy

The technique to view the inside of gastrointestinal tract has now been reduced to capsular endoscopy through the advancement of MEMS [141]. These wireless capsules consist of components such as image sensors, LED illumination devices, telemetry units for signal transmission and control electronics, all reduced to miniature sizes by microfabrication techniques. These devices have been used for diagnostic procedure of esophageal, small bowel and large bowel endoscopy [142, 143]. The first capsule-type endoscope, M2A was developed and commercialized in 2001 by Given Imaging Inc. (Israel) [144]. A limitation of these capsule endoscopes is that their movement is passive through peristaltic waves and thus their active interaction capabilities with the tissues of the digestive tract are very restricted. Presently, researches are looking into the possibility of making capsular endoscopy active through the use of micro robots [145-147]. The field of micro robotics for locomotion inside the human body is yet another interesting for the application of MEMS in biomedical technology. Different robotic mechanisms have been proposed for these active capsular endoscopes [148, 149]. Inch-worm mechanism was first proposed and tested by Dario and coworkers [150]. This concept has been improved and commercialized by the company Era Endoscopy S. r. l (Pontedera, Italy) [92].

## 6. Prosthesis

### 6.1. Neuro prosthesis

#### 6.1.1. Neuroprobes

Neural prostheses are devices that utilize electrical stimulation to activate damaged or disabled nervous system to restore function. These devices use electrical stimulation to generate action potentials in specific areas of neural population for restoring functions. Localization of these stimulations is extremely important for achieving the desired outcome. For the purpose of stimulating or recording from a neural population with reduced potential damage to the tissues, efforts have been directed to the miniaturization of neuroprobes. Miniaturization of neural probes integrated with circuitry for amplification, multiplexing, spike detection, and the wireless transmission of power and bidirectional data, are facilitating prosthetic devices for many debilitating neurological disorder [151]. Microfabrication offers the advantage of producing highly dense neuroprobe array on a single platform with desired spacing within the tissue. Integration of microfluidic channels with neuroprobes have also been investigated for drug delivery purposes [117]. The neural probe, from a biological standpoint is considered as a foreign body. Therefore, the complex aspects of biocompatibility also have been taken into consideration. For this reason, polymers such as polyimide have also been looked into for developing neural probes [152].

Neural probes for precision mapping of activity in the central nervous system have evolved from simple acute structures to complex three-dimensional electrode arrays capable of both stimulation and recording [151]. Arrays of such microelectrodes have been developed by various research groups [117, 153, 154]. For selective interfacing of the peripheral nerve, Rutten and colleagues found that an electrode separation of 120 μm gave the optimal results [155]. For the purpose of accessing more fascicles, Branner and colleagues developed a slanted array of microelectrodes with length varying from 0. 5 to 1.5 mm and it was shown to record single unit responses from mechanoreceptors [156]. Efforts have also been made to reduce the damage associated with electrode penetration, by utilizing specialized geometries, protective coatings and elastomeric microelectrode arrays [157-160].

#### 6.1.2. Regenerative electrodes

The goal behind the development of regenerative electrodes is to obtain an intimate, stable and bidirectional electrical contact with peripheral nerves. Placing sieve electrodes in the regeneration pathway of severed nerve fiber causes it to regenerate through its different holes thus enabling selective stimulation and recording of the neural bioelectrical potentials. This concept was first introduced by Marks in 1969 [161] and demonstrated by Mannard and coworkers in 1974 [162] by recording neural signals from regenerated nerves in amphibians using mechanically drilled holes. This technique was later developed and miniaturized further through micromachining techniques. Although several other groups have made efforts to obtain neural recording through regenerative electrodes, only few obtained significant in vivo results, the first of which was demonstrated by Edell [163], wherein he used a die with thin slots incorporating gold microelectrodes. These electrodes were connected to an external circuitry by means of Teflon coated silver wires. Further advancement in this field was made by the introduction of standard microfabrication technique to integrate active circuitry with the microelectrode array [164, 165].

Presently, various types of multiple holes silicon arrays, have been developed to record neural activity by interfacing nerve fibers with electrodes built around the holes [166-169]. The development of these sieve electrodes helps in providing a bidirectional interface for severed nerves of amputee's limb. For biocompatibility reasons, polyimide sieve electrodes have also been tested for the same purpose [170-173].

### 6.2. Retinal prosthesis

Of the several diseases that results in blindness, certain diseases which are limited mainly to the outer retina due to loss of photoreceptor cells have the potential of being treated using retinal prosthesis. MEMS technology provides means for developing these prosthetic devices such as ‘artificial silicon retina’ for improving the eyesight for the visually impaired. These implants stimulate the optical nerve cells mimicking the photoreceptor cells thereby producing visual sensations in the brain. The main function of retinal prosthesis is that they should be capable of detecting light reflected from surface and has to transform them into artificial stimulus such as electrical signals. Several approaches such as epiretinal, subretinal, optic nerve and cortical visual stimulations has been proposed. Generally, an epiretinal prosthesis has an image capture device, an image processing unit with a wireless transmitter, and an implant which converts these transmitted signals into a series of electrical stimulation at the remaining retinal nerve cells, whereas subretinal prosthesis mainly has an array of micro photodiodes that produces electrical current in response to light [175]. One example of subretinal prosthesis is the ‘artificial silicon retina’, a microchip containing 5,000 intrinsic photodiodes that stimulates remaining functional retinal cells [176]. Several other prototypes of silicon based on micro photodiode arrays has also been developed by other research groups [177-180]. In order to overcome the limitation brought forth by the spherical shape of retina, flexible electrodes based on polymers such as PDMS, polyimide and parylene have been investigated [181, 182]. Microfabrication technologies enable the development of these penetrating electrode arrays and integrated circuit systems that form the basis of visual prosthesis.

## 7. Microfluidics

Microfluidics involves manipulating small sample volumes of fluids in channels of the order of tens to hundreds of micrometers [183]. Due to the enormous role microfluidics plays in the development of BioMEMS devices, this topic has been treated separately in this review. Recently, microfluidic devices have also been designed for performing continuous-flow biochemical and cell-based assays [184, 185]. The ability of these microfluidic systems in mimicking the actual environment of biological systems by the use of reduced reaction volume and other resembling physiological parameters makes them more suitable for drug screening. For the above reasons, attempts to create microenvironments using microfluidic systems have been investigated [108, 186].

### 7.1. Microfluidic structure design considerations

Fundamental understanding of fluid behavior at microscale is required to design these devices. As the dimensions of the fluid system are shrunk down to micro or even nanoscale, the relative influence of physical properties of fluids such as surface tension, and fluidic resistance becomes more predominant, thus influencing its entire operation.

Due to the small dimensions of micro channels, the Reynolds's number is usually much less than 100, wherein the flow is completely laminar and thus mixing of reagents can only be done through diffusion. Various microfluidic channel geometries (T and Y shapes) have been shown to induce intermolecular mixing. In order to overcome this shortcoming and for manipulating fluid through the systems, devices such as micropumps were developed. These devices offer a number of advantages including higher throughput and faster reaction time [45, 187, 188].

### 7.2. Micropumps and Microvalves

Micropumps and microvalves constitute essential parts of microfluidic devices as they provide fine control of fluids [189]. These MEMS devices provide transport of small, accurately measured liquid quantities. Although the development of micropumps began almost 20 years ago, using them for biomedical applications was limited due to compatibility issues as well as integration difficulties. One of the earliest developed micropumps for biomedical application was an insulin delivery system developed by Smits *et al.* [190]. The actuation mechanisms used in these devices can be either mechanical or non-mechanical. Mechanical actuation mechanisms include electrostatic, piezoelectric, thermo pneumatic, SMA and bimetallic actuations. Other non mechanical actuation systems have also been studied including osmotic, Lorenz force based and electro wetting. Biodegradable osmotic micropump based on MEMS technology for long-term controlled release of large therapeutics molecules such as peptides and growth factors have also been fabricated [191].

### 7.3. Microfluidics in Tissue Engineering

Microfabrication technology has been used to fabricate microfluidic structures mimicking ‘in-vivo’ environment since cells respond to spatially and temporally organized signals in their microenvironment [106-108]. Several groups have attempted to create such structures for cell culturing [186, 192]. Mouse embryos have been grown successfully in such MEMS based microfluidic elastomeric channels [193-196]. Laminar flow streams using microfluidics has been investigated for delivering soluble substances to cells with subcellular resolution [197]. Matsue and colleagues prepared a pattern of cardiac myocytes inside a microfluidic channel and exposed it to heterogeneous flow to demonstrate the capability of this method for high-throughput drug screening and cell toxicity studies [198]. In order to overcome the biocompatibility issues, microfluidic culture system based on biocompatible materials such as PMMA, gelatin, PDMS and other polymers have been investigated [199-202]. Presently, researches are also looking into the possibility of fabricating 3D biomaterial scaffolds for providing microenvironments for cells [203-205]. Although several aforementioned microculture devices has been developed using microfabrication techniques, long term cell culturing techniques are yet to be developed that are biocompatible and efficient.

## Conclusions

In this review, we have provided a general overview of the various applications of MEMS in the biomedical field. It is evident from the growing base of research that microfabrication technologies have a played a huge role in the advancement of the biomedical tools. Several challenges still remain to be addressed, the main one being the issue of biocompatibility. However, MEMS techniques continue to give birth to devices that revolutionize the biomedical field, due to their extreme small sizes and high-volume production. By this review, we hope to have provided the reader with a general overview of the applications and the opportunities presented by MEMS in medicine. We conclude that medical applications of MEMS will have a tremendous impact in the medical care industry provided that some key technical problems are addressed and solved.

## Figures and Tables

**Figure 3:**
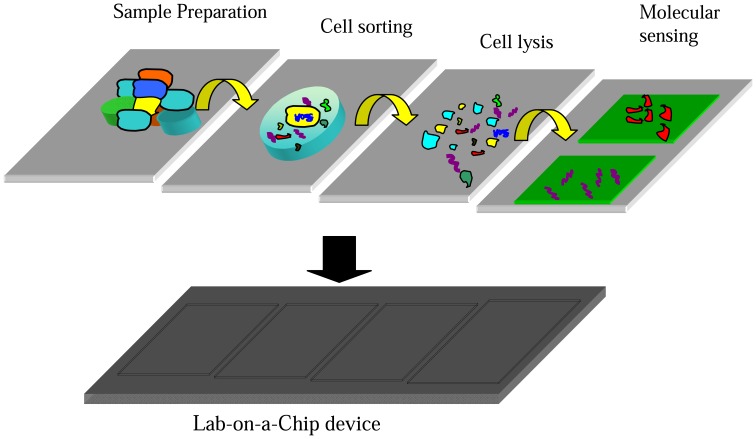
Graphical version for a Lab on a chip device for molecular diagnostics with representation of some of the processes stages. In the sample preparation stage, a specific type of cell is isolated from a cell population. Depending on the type of analyte molecule, a particular lysing mechanism is chosen to break down the cell membrane and the intra cellular materials of interest are released for analysis. If required, an analyte purification stage is also incorporated. In the sensing stage, the analyte molecules are detected and quantified.

**Figure 4:**
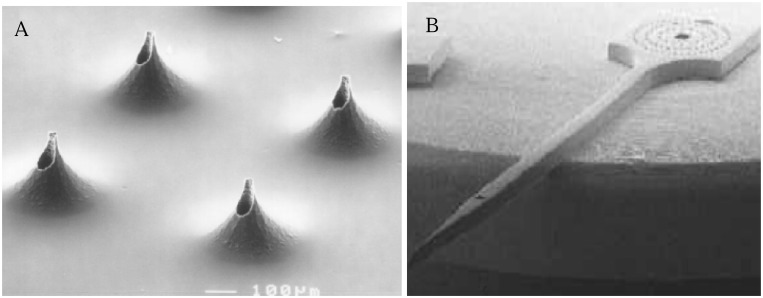
Out-of-plane (a) and in-plane (b) microneedles formed by etching silicon [120].

**Figure 5:**
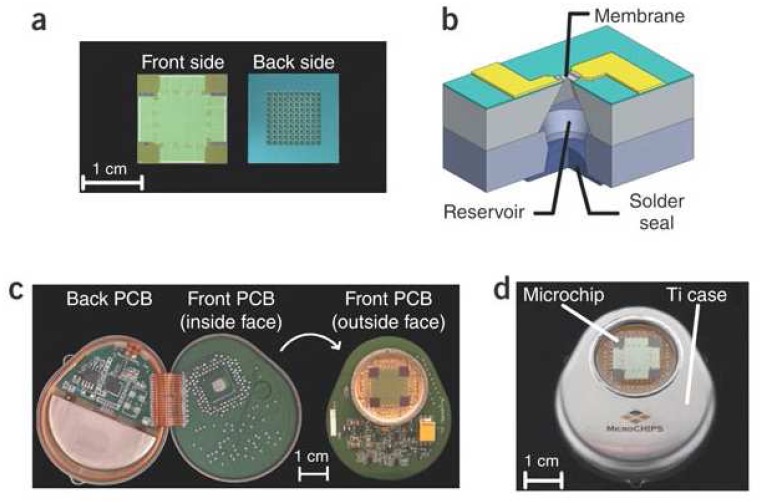
(a) Front and back of the 100-reservoir microchip. (b) Representation of a single reservoir. (c) Electronic components on the printed circuit board (PCB) in the device package. (d) The assembled implantable device. Ref [129].

**Figure 7:**
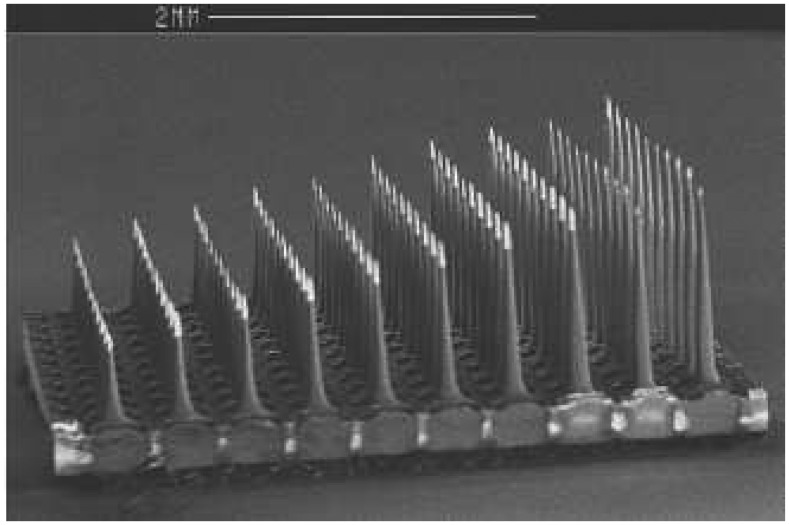
Slanted microarray developed by Branner *et al.* [156].

**Figure 8:**
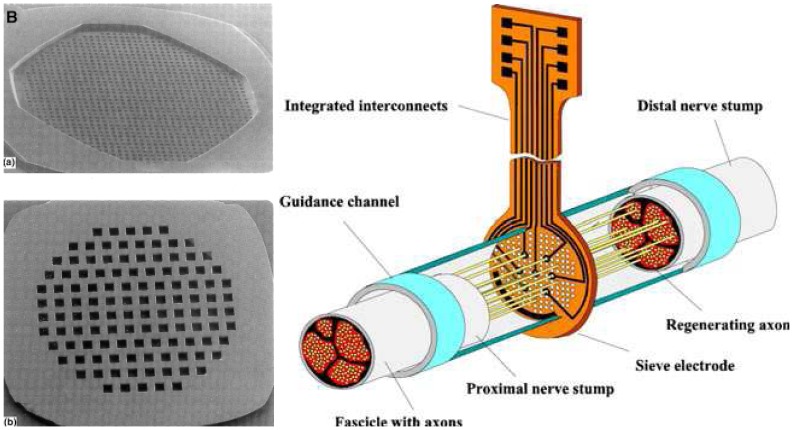
Microfabricated sieve electrodes (a &b) and a schematic of their placement in the guidance channel [173, 174].

**Figure 9:**
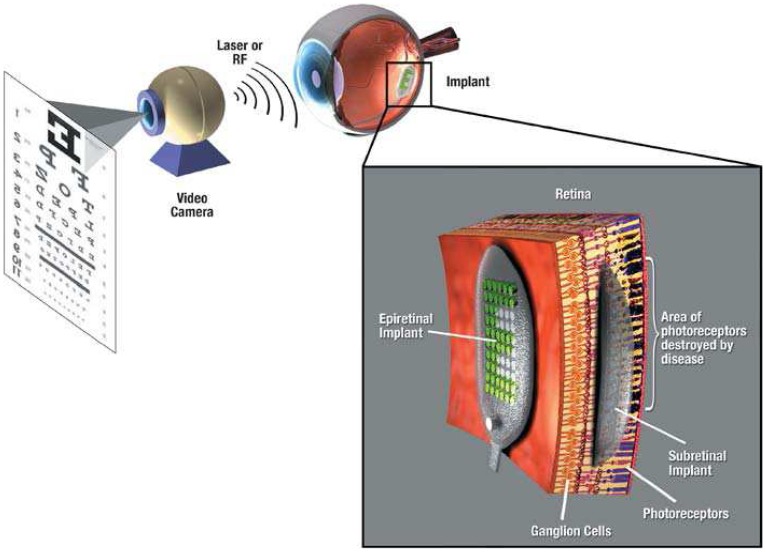
Schematic of the retinal implant for epiretinal and subretinal prosthesis [175].

**Table 1:** Various segments of diagnostics and their MEMS based examples.

**Diagnostic Sesgment**	**Purpose**	**Examples**
Clinical chemistry	Measurement of compounds or chemical reaction products in body.	Blood gas [1-3], Glucose [4-7], Ethanol [8], Cholesterol [9], uric acid [10-12], lactate[13-15], pH [16, 17] and other clinical sensors[18]
Immunochemistry	Detection of specific types of proteins using antigen/antibody chemistry	Microfabricated immunosensors [19-23]
Hematology	Characterization of blood components.	Differential blood cell counters [24-26], whole blood analysis [27], hemoglobin [28, 29] and blood ketone analysis [30]
Microbiology	Investigation of the presence of disease causing agents.	Microbial sensors [31-34]
Molecular diagnostics	Mainly focuses on diagnostics based on DNA, RNA and proteins.	DNA sensors [35-39], Immunosensors [40-42]

**Table 2:** Table shows the various stages along with the mechanisms used in MEMS devices for the development of a Lab-on-a-chip device for molecular diagnostics.

**Process stages**	**Functions**	**Mechanisms**
Cell Sorting	Isolation of cells from heterogeneous mixture of cell population	Flow cytometry, Dielectrophorosis Electrophoresis, Electro-magnetic sorting, Optical tweezers and Micro filters
Cell lysis	Disruption of cell membrane for releasing intra cellular material	Thermal, acoustic, mechanical, chemical and electrical lysing
Analytical purification	Purification/amplification of analytical molecules	PCR amplification (for nucleic acids), Purification using adhesion based technique
Molecular sensing	Detecting the presence of analyte molecules such as proteins and nucleic acids	Electrical, Mechanical, Optical Acoustic and Magnetic sensing

**Table 3:** Table shows the different phases involved in drug discovery process and contribution of MEMS technology in each phase for improved functionality.

**Process**	**Deals with**	**MEMS technologies**
Target identification	Identification of biomolecules with significant role in a specific disease.	Fabrication of DNA microarrays (in situ synthesis) [93] protein microarrays [95-101] Active microarray [94] Electronic monitoring of DNA hybridization [109-111]
Lead identification & optimization	Identification and optimization of chemical compounds that can interact with the target molecules to produce drug like effect.	Patch clamp technique [102-105] Micro electrode arrays [112] Protein and DNA microarrays
Laboratory Testing	Laboratory tests conducted on the investigational drug to see its effects both in living organism (*in vivo*) and cells (*in vitro*).	Development of microfluidic structures capable of mimicking ‘in vivo’ environment [106, 108] Cell based biosensors [113]
